# Effect of expanding nanocellulose sponge on nasal mucosal defects in an animal model

**DOI:** 10.1093/rb/rbz054

**Published:** 2020-03-03

**Authors:** Ji Won Kim, Kyungbae Woo, Jeong Mi Kim, Mi Eun Choi, Young-Mo Kim, Su-Geun Yang, Bong Sup Shim, Jeong-Seok Choi

**Affiliations:** 1 Department of Otorhinolaryngology, Inha University, College of Medicine, 27 Inhang-ro, Jung-gu, Incheon 22332, Republic of Korea; 2 Department of Chemical Engineering, Inha University, 100 Inharo, Michuholgu, Incheon 22212, Republic of Korea; 3 Department of New Drug Development, Inha University, College of Medicine, B-308, Chungsuk Bldg., 366, Seohae-Daero, Jung-Gu, Incheon 22332, Republic of Korea

**Keywords:** polyvinyl alcohol, nasal mucosa, nanocellulose

## Abstract

Nanocellulose has emerged for a wide range of applications in biomedical engineering because of its water absorption capacity, appropriate elasticity. We investigated the hemostatic and regenerative abilities of an expanding polyvinyl alcohol (PVA)-nanocellulose sponge on nasal mucosal defects. A 3 mm-diameter nasal defect was made in experimental rabbits. Rabbits were divided into four groups with control, vaseline, PVA and PVA-nanocellulose packing groups. After the defect was created, bleeding times and amounts were monitored. Packing materials were removed on experimental day (ED) 2. On ED 3, 7 and 14, histological analysis and immunohistochemical study for neutrophils were performed. Inflammatory cells were counted and epithelial thicknesses were evaluated. Bleeding amounts and times in the vaseline packing group were smaller than in the PVA groups. PVA-nanocellulose group showed less neutrophils than in the other groups on ED 7. Average epithelium thickness in the PVA-nanocellulose group was significantly smaller than in the control group at ED 7, but at ED 14, there was no significant intergroup difference. PVA-nanocellulose group had a significant lower inflammatory cell count than the control group on ED 7. PVA-nanocellulose sponge applied to nasal mucosal defects can significantly enhance mucosal regeneration during early wound healing.

## Introduction

Cellulose extracted from plants or bacteria is the most abundant polymeric resource on Earth [1]. Nanocellulose is nanostructure-formed cellulose, which is not exceeding 100 nm at least in one dimension. In nature, cellulosic materials exist as assemblies of cellulose nanofibrils in hierarchical orders [2]. Nanocellulose has various types including nanofibrils, nanofibers, nanohiskers, nanocrystals, nanorods, nanoballs and nanoplatelets [2]. Recently, cotton-derived nanocrystal have been extracted as nanocelluloses, which have shown great promise as cost-effective advanced materials for biomedical applications because of their mechanical integrities, biocompatibilities, biodegradabilities and low cytotoxicities [3]. Nanocellulose has potential use in neural tissue engineering, cartilage tissue engineering and skin wound dressings, liver, vascular and bone tissue engineering [4].

The surface functional groups of nanocelluloses can be modified to suit intended purposes. For example, 2,2,6,6-tetramethyl-piperidinyl-1-oxyl (TEMPO) oxidized cellulose nanocrystals (TOCN) produces surface carboxylic entities and preserves its fibrous crystalline structure [5]. On the other hand, oxidative degradation of amorphous portions of cellulose results in uniform, consistent properties, enhances hydrophilicity, and thus, improves dispersion in water, and enables these treated celluloses to selectively adsorb hydrophilic materials [6].

Epistaxis or nasal mucosal injuries are common—almost everyone experiences this type of injury at some time. Usually nasal cavity mucosal injuries are well controlled, but on occasion, they result in intractable bleeding and delayed wound healing [7]. Polyvinyl alcohol (PVA) is a water-processable polymer with wide commercial application due to its unique chemical and physical properties and is used to treat epistaxis [8]. PVA itself can act as not only hemostatic materials but also drug delivery due to porous structures. To improve the hemostatic effect of PVA, we designed an expanding sponge comprised of elastic nanocellulose fibers covered with a PVA matrix. To our knowledge, PVA-nanocellulose showed an intermediate pain score but minimum bleeding results in a human model [9], but there is no studies about the effect on tissue regeneration. The aim of this study was to investigate the hemostatic and regenerative abilities of this expanding PVA-nanocellulose sponge on surgically created nasal mucosal defects in a rabbit model.

## Materials and methods

### TEMPO-mediated oxidation of cellulose nanocrystals

TEMPO-mediated oxidation of cellulose nanocrystals (TOCN) was performed as previously described [10]. Briefly, TEMPO (Sigma-Aldrich) and sodium bromide (NaBr, Sigma-Aldrich) were added to 100 ml of distilled water and mixed with a magnetic stirrer at room temperature, and then ∼1 g of natural cellulose was added to this TEMPO/NaBr solution. After the cellulose had loosened, NaClO (sodium hypochlorite) was added to the mixture to convert primary cellulose C6 hydroxyls into carboxylate groups. To maintain a pH of 10–11, 1 M NaOH was intermittently dropped into the mixture to promote the reaction. After oxidation, salt free cellulose filter cake was obtained by centrifugation and vacuum filtration, dried in an oven at 40°C and then dispersed in distilled water using an ultrasonic probe three times. The solution obtained was then centrifuged to remove the metallic particles from the probe to obtain a TOCN solution as a clear supernatant.

**Figure 1:**
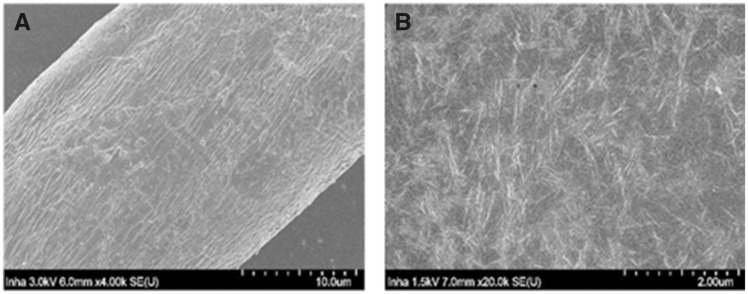
Scanning electron microscope (SEM) images of cotton fiber and cotton fiber TEMPO-mediated oxidation of cellulose nanocrystals (TOCN): (**A**) cotton fiber; (**B**) cotton fiber TOCN.

Electron microscope (HR-SEM, SU8010, Hitachi) at 3 kV accelerating voltage and Fourier-transform spectroscopy was used for checking TOCN morphology and functional group change. Figure 1 shows the morphology of cotton cellulose and TOCN extracted from cotton at different magnification. Both cotton cellulose and TOCN are rod-like forms, having long length compared with width. Figure 1A shows the cotton cellulose which is composed of microfibrils and Fig. 1B shows the TOCN image which is nanosized whisker-like crystalline nanofiber and it shows that cotton fiber (100% premium cotton pads) was successfully nanosized by tempo-oxidized.

### Preparation of a PVA sponge

Prior to preparing PVA sponge, PVA (MW = 125 000, Sigma-Aldrich) was dissolved in distilled water and heated in a water bath at 80°C to prepare the 10 wt% PVA aqueous solution. For making bubble and preparing a crosslinking reaction, the solution was mixed with triton X-100 and 37% formaldehyde (Merk) by a homogenizer. About 35% HCl was added directly to the frothed solution and remixed by a homogenizer. The final frothed solution was poured to a pre-heated conical tube and transferred into the 65°C oven for cross-linking. After 6 h, solid foam was washed by tap water to remove unreacted residues. The solid foam was put into a considerable quantity of distilled water, and then the distilled water was heated 1 h for entirely removing pore trapped residues (especially Triton-X 100) by using vaporized DI water bubble. In order to quench unreacted formaldehyde, the sample was put into 1 wt % glycine and stirring overnight. The solid foam was washed by a great quantity of DI water and dried 3 days at room temperature.

### Preparation of a PVA-TOCN sponge

For preparing PVA-TOCN sponge, PVA was dissolved in TOCN solution and heated in a water bath at 80°C to prepare 10:1 ratio PVA-TOCN aqueous solution. For making bubble and preparing crosslinking reaction, the solution was mixed with triton X-100 and 37% formaldehyde by a homogenizer. About 35% HCl was added directly to the frothed solution and remixed by a homogenizer. The final frothed solution was poured to a pre-heated conical tube and transferred into the 65°C oven for cross-linking. After 6 h, solid foam was washed by tap water to remove unreacted residues. The solid foam was put into a great quantity of DI water, and then the DI water was heated 1 h for fully removing pore trapped residues (especially Triton-X 100, Sigma-Aldrich) by using vaporized distilled water bubble. In order to quench unreacted formaldehyde, the sample was put into 1 wt % glycine and stirring overnight. The solid foam was washed by a great quantity of distilled water and dried 3 days at room temperature.

### Animals

**Figure 2:**
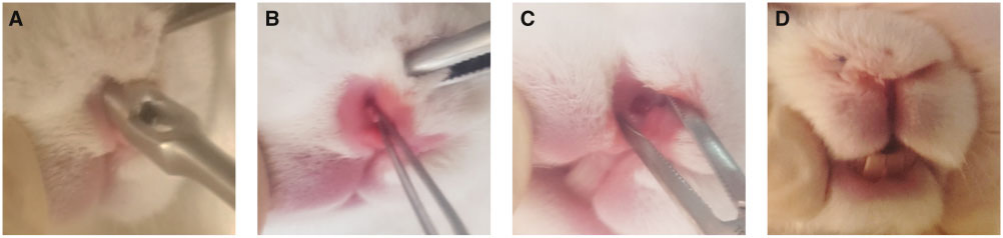
Rabbit model of nasal mucosal injury. (**A**) A circular punch was used to create nasal mucosal defects, (**B**, **C**) a circular (3 mm diameter), full thickness mucosal defect in the right nasal vestibule and (**D**) packing materials were sutured with nylon 4-0 to the nasal wall.

All experiments conducted on animals were carried out according to the guidelines for animal experiments issued by Inha University School of Medicine. Study approval was obtained from the Institutional Animal Care and Use Committee of Inha University (INHA 180402-550) beforehand. Twelve mature domesticated rabbits (New Zealand white, 2–2.5 kg) were used in the study. Animals were randomly assigned to one of four groups: group I (no treatment), group II (vaseline), group III (PVA) and group IV (PVA-nanocellulose). Each animal received an intramuscular injection consisting of Zoletil^®^ (Tiletamine/zolazepam, 10 mg/kg) and Rompun^®^ (2% xylazine injection, 100 mg/kg) at a ratio of 1:2 for general anesthesia. A 3 mm-diameter circular mucosal defect was made in the nasal cavity floor of each rabbit. To preventing migration, packing materials were sutured with nylon 4-0 to the nasal wall (Fig. 2).

Animals in group II received vaseline gauze packing, those in group III received PVA packing and those in group IV received PVA-nanocellulose packing. Animals in group I (controls) received no treatment. Bleeding times were noted after surgery, and animals were monitored for 10 min to check for rebleeding. Bleeding amounts were determined by weighting packing material before and after bleeding had stopped. Animals were sacrificed at 3, 7 and 14 days after injury. Histological sections were examined for inflammatory cell infiltration by hematoxylin and eosin (H&E) staining. Fibrosis was evaluated using Masson’s trichrome-stained sections. The immunohistochemical study was performed using neutrophil antibody (sc-59376, Santa Cruz, CA, USA). A blinded examiner evaluated three random fields per section, and inflammatory cell numbers were determined by counting pixels using Metamorph software (Molecular Devices Corporation, Sunnyvale, CA, USA). Subepithelial and epithelial thicknesses were measured.

### Statistical analysis

The statistical analysis was performed using the GraphPad Prism 8 package (GraphPad Software Inc., La Jolla, CA, USA). Intergroup comparisons were performed by one-way analysis of variance (ANOVA) followed by Tukey’s multiple comparison test, and paired group comparisons were performed using two-way ANOVA followed by Bonferroni’s *post hoc* test. Statistical significance was accepted for *P*-values < 0.05.

## Results

### Effects on hemostasis

**Figure 3:**
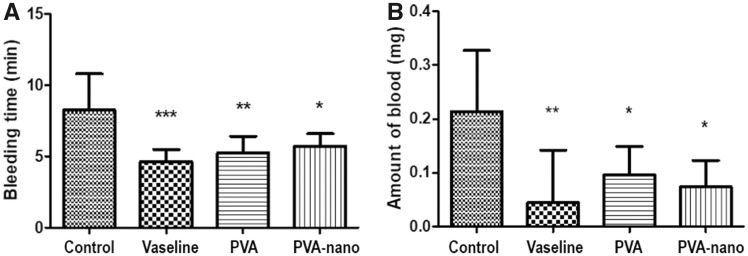
Bleeding times and amounts. Results are presented as means ± SDs. PVA, polyvinyl alcohol; PVA-nano, polyvinyl alcohol-nanocellulose; SD, standard deviation. *Compared with the control group. *P < 0.05; **P < 0.01; ***P < 0.001.

Animals in the vaseline group had the lowest bleeding time; no difference was observed between the PVA and PVA-nanocellulose groups (Fig. 3A). In terms of the amount of bleeding, the PVA and PVA-nanocellulose groups showed less blood loss (0.096 ± 0.052 mg and 0.074 ± 0.048 mg, respectively) than the control group (Fig. 3B).

### Wound healing effect

**Figure 4:**
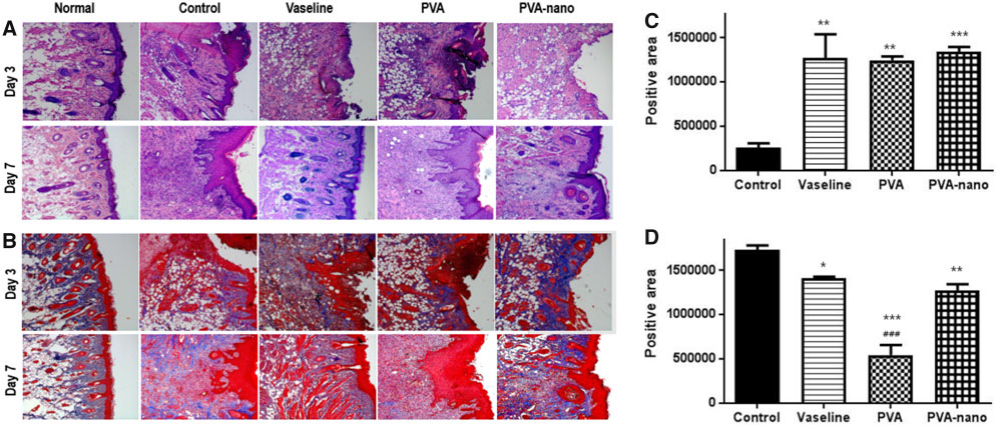
Histologic findings of nasal mucosa at 3 and 7 days postoperatively. (**A**) H&E and (**B**) MT staining (×200). (**C**) Differences areas of fibrosis in the four groups on day 3. (**D**) Differences areas of fibrosis in the four groups on day 7. H&E, hematoxylin and eosin; MT, Masson’s Trichrome. *Compared with the control group; ^#^compared with the vaseline group. *P < 0.05, **P < 0.01, ***P < 0.001; ^###^*P* < 0.001

Group histopathological ﬁndings are summarized in Fig. 4. In all animals, H&E staining showed moderate to severe inflammation on ED3 (3 days postoperatively; Fig. 4A). Figure 4C showed the difference of the fibrosis positive areas in all groups. Group II (vaseline), group III (PVA) and group IV (PVA-nanocellulose) demonstrated the significantly higher positive area comparing the group I (no treatment), respectively. Amounts of collagen and granulation tissue were significantly greater in the vaseline and PVA groups than in the control group (*P* < 0.05) (Fig. 4B). However, on ED7, amounts of collagen and granulation tissue were significantly lower in the vaseline and PVA groups than in the control group and fibrotic area was lowest in the PVA group (*P* < 0.001) (Fig. 4D). On ED14, all mucosal defects had completely healed (data not shown).

### Anti-inflammatory effect

**Figure 5:**
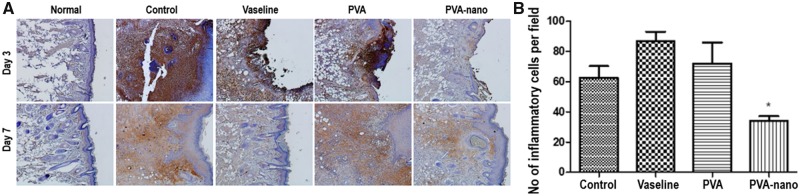
Anti-inflammatory properties of PVA-nanocellulose (**A**) immunohistochemical staining for neutrophils (×200). (**B**) Analysis of inflammatory cell counts on ED7. *Compared with the control group. *P < 0.05.

Immunohistochemical staining for neutrophil showed significantly lower inflammatory cell numbers in the nasal mucosa of the PVA-nanocellulose group than the other three groups on ED7 (*P* < 0.05) (Fig. 5A). Quantitative analysis of inflammatory cells also showed the PVA-nanocellulose group had a significant lower inflammatory cell count than the control group on ED7 (*P* < 0.05) (Fig. 5B).

### Mucosal thickness

Loss of normal epithelium was observed during early postoperative period within ED7. Thicknesses of whole mucosa and epithelia were increased with time during the early postoperative period. The average epithelial thicknesses in the four groups were 13 ± 2.16, 14.93 ± 3.09, 14.03 ± 1.93 and 35 ± 5.39, on ED3, 126.6 ± 13.99, 33.2 ± 8.33, 135.5 ± 23.12 and 41.6 ± 6.14 on ED7 and 22.68 ± 6.14, 16.93 ± 0.83, 21.47 ± 4.66 and 26.40 ± 6.21 on ED14, respectively (Table 1). Epithelial thickness in the PVA-nanocellulose group did not change significantly with time.

**Table 1:** Group epithelial thicknesses at 3, 7 and 14 days postoperatively

Group	Epithelial thickness (µm)		
	Day 3	Day 7	Day 14
Control	13.00 ± 2.16	126.6 ± 13.99	22.68 ± 4.30
Vaseline	14.93 ± 3.09	33.2 ± 8.33*	16.93 ± 0.83
PVA	14.03 ± 1.93	135.5 ± 23.12	21.47 ± 4.66
PVA-nanocellulose	35.00 ± 5.39	41.60 ± 6.14***	26.40 ± 6.21

PVA, polyvinyl alcohol.

* *P *<* *0.05 compared with the negative control group.

** *P *<* *0.05 compared with the PVA group.

## Discussion

For nasal mucosal injuries, electro or chemical coagulation and packing treatment are regarded as the first-line treatments to achieve hemostasis. However, a significant percentage of patients experience delayed mucosal healing and rebleeding [10]. Oxidized regenerated cellulose is a widely available surgical hemostatic material and is even used to treat luminal gastrointestinal bleeding [11, 12]. This is the first animal study to evaluate the hemostatic and regenerative effects of PVA-nanocellulose sponge for the treatment of nasal mucosal defects.

Bleeding time reflects primary hemostasis [13], and in the present study, group bleeding times were not significant different, though the vaseline group showed least blood loss, which we attributed to the hydrophobic nature of vaseline. Based on the previous literature [4, 14], satisfactory hemostatic performance of nanocellulose is enhanced with the combination of other materials including thrombin, argon plasma, etc. We confirmed the hemostatic ability of nanocellulose by loading in PVA which was used as conventional epistaxis treatment materials. Hemostasis is the first step toward wound healing and facilitates blood clotting on wound surfaces [13]. Basu *et al.* reported nanofibrillated cellulose efficiently initiated coagulation due to rapid onset of clot formation through the complement system [13]. In the present study, PVA-nanocellulose as well as PVA sponges showed better hemostasis than was observed in the non-treated controls.

Weber *et al.* [12] reported four phases of sinus surgery wound healing in man, that is, hemostasis (postoperative days 7–12), granulation tissue formation (postoperative weeks 2–4), an edematous phase and a normalization phase (postoperative weeks 12–18). Interestingly, our results showed normalization occurred 2 weeks postoperatively, which may have been due to the small size (3 mm) of the nasal mucosa defects. Khalmuratova *et al.* [15] described the wound healing process in a rat nasal mucosal defect model, and noted edematous epithelium and neutrophil infiltration at 2 days postoperatively, monocyte infiltration and granulation at 5 days, increased subepithelial fibrosis and epithelial thickness at 14 days, goblet and ciliated cell regeneration from 14 days and restoration to near normality at 28 days. Our results showed the recovery of epithelial thickness within 14 days after nasal mucosal injury, and that was in line with previous similar study designed study [16]. On the other hand, this present study demonstrated the collagen fiber density and subepithelial fibrosis were greater in vaseline, the PVA and PVA-nanocellulose than in the control group on experimental day (ED) 3. However, on ED7, the PVA and PVA-nanocellulose groups showed less granulation than on ED 3, whereas collagen fibers started to collect in the control group, which suggested the PVA-nanocellulose helped mucosal regeneration during the early period, especially with respect to hemostasis and granulation tissue formation. During wound healing process, over deposition of collagen occurs to regenerate the injured mucosa [16].

Healing after tissue injury involves four overlapping processes, namely, hemostasis, inflammation, proliferation and remodeling. The factors that related inflammation include reduced tissue nutrition, increased oxidative stress and increased tissue destruction [17]. We found neutrophil counts in the PVA-nanocellulose groups were lower comparing with control and PVA only group. That suggested that nanocellulose can have an anti-inflammatory effect during the healing process. Samulin *et al.* [18] concluded cellulose nanocrystal exposure may result in dysregulations of macrophage activation and function, which are critically required for inflammatory responses in lung. Wound healing is a highly organized and complex process that includes inflammation, re-epithelialization, matrix deposition and tissue remodeling and is regulated by a wide variety of cytokines and growth factors through the recruitment of inflammatory leukocytes, fibroblasts and epithelial cells [19]. Our findings suggest the lesser inflammation reaction caused by PVA-nanocellulose reduced lower growth factor release and resulted in less epithelial thickening.

The present study has a number of limitations that warrant consideration. First, because of the preliminary nature of the present study the number of samples was small. Second, we did not consider potential physical or chemical interactions between PVA and nanocellulose. Third, we did not evaluate long-term effects on wound healing. Long-term histological study will be needed especially if we are testing this on humans.

## Conclusion

We evaluated the ability of the nasal mucosal regeneration in PVA-nanocellulose sponge. To our knowledge, this is the first animal study to nanocellulose, which is widely used in industry, technology, biotechnology and medicine, including tissue engineering, loading PVA to nasal mucosal damage. PVA-nanocellulose sponge can enhance mucosal regeneration of nasal mucosal defect during the early stage of wound healing from the perspective of anti-inflammation. We anticipate that this novel biomaterial will become a new treatment for nasal mucosal injury. However, further safety aspects on the nanocellulose should be taken into account while using them as direct packaging materials.

## Acknowledgements

This research was supported by the Basic Science Research Program, the Medical Research Center (MRC) through the National Research Foundation of Korea (NRF) by the Korean government (MOE and MSIT) (NRF-2016R1D1A1A02937416, NRF-2017R1A6A3A11027865, NRF-2017R1D1A1B03030819, 2018R1A6A1A03025523 and NRF-2014R1A5A2009392) and also supported by Civil Military Co-Technology Development Program (16-CM-SS-07) from Civil Military Technology Cooperation Center. B.S.S. acknowledges the funding supports by NRF-2017R1A2B4012736 and NRF-2018K1A3A1A32055149.

*Conflict of interest statement*. None declared.
